# *In Silico* Design of *phaCAB* Expression Constructs for Cellulolytic Hosts Toward Hemp Hurd Valorisation and Polyhydroxybutyrate Biosynthesis

**DOI:** 10.3390/molecules31152729

**Published:** 2026-08-06

**Authors:** Ziningi Rosebud Myeni, Sani Gumede, Nomfundo Ntombela, Farai Dziike, Nirmala Deenadayalu

**Affiliations:** 1 Department of Chemistry, Faculty of Applied Sciences, Durban University of Technology, Steve Biko Campus, 70 Steve Biko Road, Berea, Durban 4001, South Africa; 2 Technology Innovation Agency Bioprocessing Platform, 1 Dickens Road, 28 Warhist Street, Umbongwintini Industrial Complex, Umbongwintini, Durban 4120, South Africa

**Keywords:** hemp hurds, consolidated bioprocessing, circular bioeconomy, polyhydroxybutyrate, synthetic biology, waste valorisation

## Abstract

Hemp hurds (HHs), an underutilised lignocellulosic biomass (LB) from agricultural waste, offer potential for bioconversion into high-value bioproducts within a circular bioeconomy. Building on prior work involving magnetic nanoparticle-immobilised cellulase hydrolysis of pretreated HH, this study computationally designed candidate *phaCAB* expression constructs for cellulolytic hosts toward future polyhdroxybutyrate (PHB) production. The objective was to evaluate, *in silico*, the feasibility of introducing the *phaC1*, *phaA* and *phaB1* genes from *Cupriavidus necator* H16 (*C. necator*) (assembly GCA_000009285.2; loci H16_A1437–H16_A1439) into the cellulolytic hosts *Clostridium thermocellum* DSM 1313 (*C. thermocellum*) and *Trichoderma reesei* RUT C-30 (*T. reesei*). Coding sequences were retrieved and translated individually and host-specific expression compatibility was assessed via codon adaptation index, effective number of codons, GC content and rare-codon frequency/clustering. Host-specific architectures were designed: three independent *tef1*-promoter cassettes with fungal Kozak contexts and *cbh1* terminators for *T. reesei* (TrePHB3 integration construct), and a single *groEL*-promoter operon with graded ribosome-binding sites for the pIKM1-based *C. thermocellum* construct (pCtPHB1); *Escherichia coli* BL21 (DE3) (*E. coli*)/pET-24a(+) served only as an intermediate assembly platform. Clustal Omega alignment, virtual plasmid assembly and simulated restriction digestion (SnapGene) confirmed preservation of the open reading frames and expected fragment sizes. ProtParam analysis indicated instability indices below 40 and negative GRAVY values for *PhaC*, *PhaA* and *PhaB*, indicating overall hydrophilic character. This computational framework links HH valorisation, cellulolytic Consolidated Bioprocessing (CBP) hosts and PHB pathway design within a conceptual biorefinery, supporting future integrated biomass-valorisation platforms; it is computational only and does not demonstrate transformation, expression, PHB accumulation or biomass conversion, which require experimental validation.

## 1. Introduction

Environmental pollution associated with synthetic plastics has become an increasing concern due to their persistence and limited biodegradability. Polyhydroxybutyrate (PHB) is a microbial polyester belonging to the polyhydroxyalkanoate (PHA) family and is one of the most studied biodegradable polymers. PHB exhibits thermoplastic properties comparable to polypropylene, including average molar mass, high crystallinity (55–80%), melting point up to 180 °C, and high tensile strength, indicating similar fundamental properties [1,2]. Due to its biodegradability and biocompatibility, PHB has attracted increasing attention for applications in packaging materials, biomedical devices, agricultural films and environmentally sustainable plastics. However, large-scale PHB production remains economically challenging due to the high cost of carbon substrates and fermentation processes. Consequently, identification of low-cost LB feedstocks capable of supplying fermentable sugars remains a critical objective for sustainable PHB production.

Lignocellulosic biomass (LB) derived from HH, an agricultural waste, represents a promising substrate for biopolymer production due to its abundance, low cost, and carbon-neutral profile. HHs are the woody inner cores remaining after fibre extraction from industrial hemp and represent an underutilised LB with potential for biorefinery applications.

HHs offer a renewable alternative that addresses agricultural waste disposal issues while promoting a circular economy [3]. For renewable feedstocks to be industrially viable, they must be cost-effective, scalable and capable of meeting global material demands [4].

The transition towards a circular bioeconomy requires the development of technologies capable of converting low-value agricultural residues into high-value bioproducts. The LB composition of HH consists of high carbohydrate content (32.6–51.1% cellulose, 10.6–16.6% hemicellulose, 18% pectin) and reduced lignin presence (3.7–20.0%) [5]. This makes HH a promising feedstock for biofuel, chemical and biodegradable polymer production. The use of HH aligns with circular economy principles through promoting resource efficiency, reducing agricultural waste accumulation and supporting the replacement of fossil-derived materials with renewable alternatives [6].

The valorisation of HH into PHB presents an opportunity to integrate waste management, sustainable material development and biotechnology within a single circular bioeconomy framework [2,6]. Although bioproducts made from LB meet two of these criteria, their cost-effective production is currently lacking because of the challenges in hydrolysis and converting the sugars locked within the LB, contributing an estimated 20–30% of total production costs [7].

Despite its favourable carbohydrate composition, efficient use of HH remains constrained by structural and physicochemical barriers that limit enzymatic accessibility. Previous work demonstrated that HHs contain substantial polysaccharide content (53.4%), moderate lignin content (20.8%) and a crystallinity index of 40.2%, confirming their suitability as a lignocellulosic feedstock for bioconversion processes [3]. Subsequent pretreatment studies identified ultrasound-assisted sodium hydroxide pretreatment as the most effective strategy for structural disruption, resulting in a crystallinity index of 65.8%, reduced lignin-associated functional groups and improved cellulose accessibility [8]. Collectively, these findings established a foundation for the enzymatic conversion of HH-derived carbohydrates into value-added bioproducts.

In HH, the matrix of hydrogen bonding within crystalline cellulose and the hydrophobic interactions with lignin cause enzymatic conversion to be inefficient [9]. Bioconversion of polysaccharides embedded in cellulose structure requires disruption of crystalline hydrogen bonding and hydrophobic cross-linking between cellulose, hemicellulose, and lignin [10]. Such a structure increases pretreatment costs, which are necessary to overcome structural barriers, constituting at least 20% of the entire production cost [7].

The crystalline nature of cellulose represents one of the primary barriers to efficient enzymatic hydrolysis. Extensive intra- and intermolecular hydrogen bonding reduces enzyme accessibility to glycosidic linkages, thereby limiting cellulose depolymerisation. In addition, lignin acts as a physical barrier surrounding cellulose microfibrils and may adsorb cellulolytic enzymes non-productively, reducing hydrolysis efficiency. Hemicellulose further contributes to structural complexity by forming a heterogeneous matrix that reinforces interactions between cellulose and lignin.

These physicochemical characteristics collectively reduce the accessibility of cellulolytic enzymes to fermentable carbohydrate fractions and often necessitate pretreatment prior to hydrolysis. Consequently, efficient LB conversion requires integrated strategies capable of improving enzyme accessibility, enhancing hydrolysis performance and maximising sugar release. Addressing these limitations is essential for the development of economically and environmentally sustainable biomass-valorisation pathways capable of converting agricultural residues such as HH into value-added bioproducts.

The conversion of LB into fermentable sugars remains one of the major challenges limiting the economic and technical feasibility of biorefineries. Enzymatic hydrolysis using cellulase enzymes is widely regarded as an environmentally friendly approach for cellulose depolymerisation because it operates under mild reaction conditions and minimises the formation of inhibitory by-products. Free cellulase systems have been extensively investigated for LB conversion; however, their industrial application is often constrained by limited operational stability, challenges associated with enzyme recovery and the requirement for repeated enzyme addition during successive hydrolysis cycles.

Enzyme immobilisation has emerged as a promising strategy to overcome these limitations. Immobilised cellulases can exhibit improved operational stability, enhanced resistance to environmental conditions and the ability to be recovered and reused across multiple hydrolysis cycles. These characteristics may contribute to reduced enzyme consumption and improved process efficiency during biomass conversion. Furthermore, immobilisation can facilitate sustained enzymatic activity while maintaining hydrolytic performance, making it an attractive approach for lignocellulosic biorefinery applications.

For HH valorisation, immobilised cellulase systems offer the potential to improve cellulose hydrolysis efficiency and increase the release of fermentable sugars required for downstream bioconversion processes. Building upon the feedstock characterisation and pretreatment studies, previous work by the authors demonstrated successful immobilisation of *T. reesei* cellulase onto amine-functionalised magnetic nanoparticles (MNP) with approximately 89% retained enzymatic activity. The immobilised cellulase system generated 88–91% of the glucose produced by free cellulase across multiple enzyme loadings and retained approximately 64% of its initial hydrolytic performance after five reuse cycles. These findings demonstrated the feasibility of generating fermentable sugar streams from pretreated HH using recyclable cellulase nanobiocatalysts. This route, in which pretreated HHs are hydrolysed by immobilised cellulase and the recovered sugars are then fermented, is a separate hydrolysis and fermentation (SHF) configuration rather than strict CBP. The present study builds conceptually on this biomass valorisation pathway by computationally designing candidate *phaCAB* expression constructs for cellulolytic consolidated-bioprocessing hosts, toward future polyhydroxybutyrate production.

The bioconversion of HH into valuable bioproducts entails several steps, such as biomass pretreatment, enzymatic hydrolysis and fermentation [11]. There are three primary methods to convert HH into fermentable sugars and fermentation to bioproducts: (i) separate hydrolysis and fermentation (SHF), (ii) simultaneous saccharification and fermentation (SSF) and (iii) consolidated bioprocessing (CBP), which occurs in one bioreactor and is the least researched method [12]. The integration of these steps is a necessity for economic and environmental reasons. CBP has gained attention because it integrates biomass hydrolysis and fermentation into a single process step, thereby reducing operational cost. Consequently, synthetic biology and metabolic engineering approaches have emerged as key strategies for integrating biomass degradation and product biosynthesis within single microbial platforms.

While previous studies have investigated microbial PHB production from simple carbon substrates, few studies have explored the conceptual integration of LB-degrading microorganisms with heterologous PHB biosynthetic pathways within a CBP framework. For PHB production from LB, CBP relies on engineered microbial cell factories capable of both LB degradation and product formation in a single process. However, developing highly efficient microorganisms for LB conversion remains challenging. Current genetic and metabolic engineering strategies focus either on engineering cellulolytic microorganisms to produce value-added products or modifying product-producing microorganisms to express cellulolytic enzymes, thereby enhancing simultaneous enzyme hydrolysis and fermentation of LB [11,12].

The integration of biomass pretreatment, enzymatic hydrolysis, consolidated bioprocessing and PHB biosynthesis represents a potential strategy for converting LB from HH into biodegradable polymers within a circular bioeconomy framework.

Among the microorganisms investigated for CBP applications, cellulolytic fungi and thermophilic bacteria represent attractive candidates due to their intrinsic biomass-degrading capabilities and potential for heterologous pathway engineering.

*T. reesei* is a filamentous fungus with a high capacity to produce cellulolytic enzymes, including endoglucanases, cellobiohydrolases and β-glucosidases that act synergistically to hydrolyse cellulose into fermentable sugars. Endoglucanase cleaves internal β-(1 → 4)-glycosidic bonds within cellulose chains, reducing polymer length and creating new chain ends. Cellobiohydrolase (exoglucanase) then hydrolyses cellulose from these chain ends, releasing cellobiose as the primary product. Finally, β-glucosidase converts cellobiose into glucose. Together, these enzymes depolymerise cellulose into fermentable glucose [13,14]. This makes *T. reesei* a key organism in industrial applications for biomass degradation and biofuel production and in the textile, paper and food industries.

*C. thermocellum* is a thermophilic anaerobic bacterium isolated from soil with high potential for LB degradation [15]. It is a Gram-positive bacterium that produces cellulosome enzymes known for degradation of both crystalline cellulose and hemicellulose [16]. This is a unique feature for *C. thermocellum*, enabling direct hydrolysis of biomass. *C. thermocellum* produces a highly efficient cellulosome capable of solubilising crystalline cellulose and hemicellulose. *C. thermocellum* encodes a complex cellulosome with various catalytic subunits, including endoglucanases, exoglucanases, xylanases and additional hemicellulases, along with carbohydrate-binding modules (CBM3) for substrate attachment. The cellulosomes in *C. thermocellum* exist both as cell-bound and unbound complexes, in addition to free, individual enzyme systems. This supports the effective solubilisation and decomposition of LB into reducing sugars, primarily cellodextrins and cellobiose with smaller amounts of glucose [17]. *C. thermocellum* preferentially metabolises cellodextrins, while any contribution of glucose utilisation to heterologous PHB production remains to be experimentally established. Although *C. thermocellum* possesses highly efficient biomass-degrading capabilities, *C. thermocellum* is not a recognised native PHB-production host. Consequently, the introduction of heterologous PHB biosynthetic pathways represents a promising strategy for coupling LB degradation with biodegradable polymer production within a CBP framework.

Engineered CBP hosts require expression systems compatible with the physiological and genetic characteristics of the target microorganism [18,19]. Synthetic biology provides a platform for introducing heterologous pathways into cellulolytic microorganisms, enabling conversion of LB-derived sugars into products beyond their native metabolic range. In this study, host-specific expression designs were selected to provide a theoretical framework for evaluating the compatibility of the PHB biosynthetic genes with fungal and bacterial cellulolytic hosts. Computational construct design allows preliminary assessment of gene organisation, insert orientation and vector architecture before experimental implementation and is increasingly used in metabolic engineering to support rational strain development.

To support the computational design, host-specific platforms were selected for *E. coli*, *T. reesei* and *C. thermocellum*. The pET-24a(+) vector was used only as an intermediate assembly platform in *E. coli* BL21 (DE3) because of its widespread use in recombinant work. For *T. reesei*, a dedicated fungal expression architecture based on the constitutive *tef1* promoter and *cbh1* terminator was designed rather than a heterologous yeast vector, so that the three biosynthetic genes could be expressed as individual, host-compatible cassettes. For *C. thermocellum*, the shuttle vector pIKM1 was retained as the backbone and supplemented with a thermophile-compatible *groEL* promoter, individual ribosome-binding sites and a transcription terminator. Successful PHB biosynthesis requires intracellular acetyl-CoA and reducing equivalents in the form of NADPH; both hosts possess central carbon pathways capable of generating these precursors from LB-derived sugars, although the extent to which carbon flux can be redirected towards PHB accumulation remains to be evaluated experimentally.

The valorisation of LB into biodegradable polymers is constrained by the limited availability of microbial platforms capable of simultaneously degrading biomass and synthesising value-added products. Previous studies have separately demonstrated PHB production from lignocellulosic hydrolysates, heterologous expression in *T. reesei*, genetic manipulation of *C. thermocellum* and recombinant expression of PHB biosynthetic pathways. Recombinant *E. coli* has produced PHB from hydrolysates derived from non-recyclable fibre rejects under a separate hydrolysis and fermentation configuration, and engineered *C. necator* has been reported to convert mixed sugars from real lignocellulosic hydrolysates [20,21]. These studies confirm that lignocellulosic hydrolysates can support polymer production but do not link biomass degradation and PHB synthesis within a single cellulolytic host, nor do they connect the process to a defined hemp-hurd valorisation sequence. The comparison in Table 1 positions the present work against these categories of prior study.

**Figure 1:**
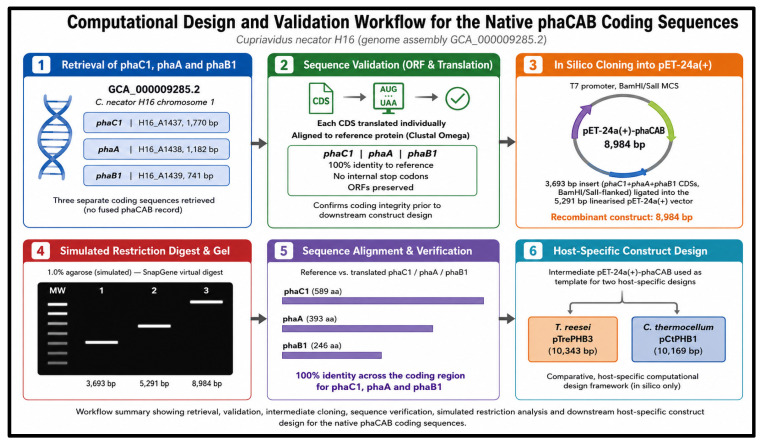
Computational design and validation workflow for the native *phaCAB* coding sequences from *Cupriavidus necator* H16. The individual *phaC1* (H16_A1437), *phaA* (H16_A1438) and *phaB1* (H16_A1439) coding sequences were retrieved from genome assembly GCA_000009285.2, translated and validated as separate open reading frames, assembled *in silico* into the intermediate pET-24a(+)-*phaCAB* construct and subsequently evaluated by sequence alignment and simulated restriction analysis before host-specific construct design for *T. reesei* and *C. thermocellum*.

All analyses were performed computationally and did not imply experimental validation. Rather than focusing solely on cloning feasibility, this work establishes a conceptual biomass-valorisation platform linking agricultural waste utilisation, enzymatic biomass conversion, consolidated bioprocessing and biodegradable polymer production. By combining fungal and bacterial cellulolytic architectures with the PHB biosynthetic pathway, the study provides a foundation for future development of engineered microbial systems capable of converting hemp hurd biomass into sustainable bioplastics within a circular bioeconomy as illustrated in Figure 1.

The contribution of this study is therefore not the first computational cloning of the *phaCAB* genes. It is the development of a comparative, host-specific computational design framework that is conceptually and positionally linked to an established HH valorisation sequence, spanning feedstock characterisation, pretreatment and immobilised-cellulase hydrolysis, with candidate PHB expression architectures for two mechanistically distinct cellulolytic hosts. This study does not itself analyse HH hydrolysate composition, substrate uptake, inhibitor tolerance, carbon flux from HH sugars, or PHB production from HH; it should therefore be read as a candidate construct-design framework positioned within a future HH valorisation pathway, rather than a validated hemp-hurd bioconversion platform. Prior *phaCAB* engineering has centred on non-cellulolytic chassis, including promoter- and construct-design studies in *E. coli* [22,23] and heterologous expression in transgenic plants [24]; for the *C. thermocellum* host specifically, previous work has largely focused on identifying genetic-engineering targets and enabling host manipulation rather than establishing a heterologous PHB pathway [25]; the representative studies considered here do not link *phaCAB* expression to a cellulolytic CBP host or to a defined lignocellulosic waste stream such as HH. The framework remains computational and requires experimental validation. To support this concept, an *in silico* workflow was developed to evaluate the design feasibility of introducing the individual *phaC*, *phaA* and *phaB* genes into *T. reesei* and *C. thermocellum*, using *E. coli* as an intermediate assembly host. All analyses were computational and did not imply experimental validation.

## 2. Materials and Methods

### 2.1. Retrieval and Validation of the phaCAB Genes

The native *phaC1* (H16_A1437), *phaA* (H16_A1438) and *phaB1* (H16_A1439) coding sequences of the *C. necator* PHB biosynthetic operon were retrieved from the annotated genome assembly GCA_000009285.2 (chromosome 1 accession AM260479.1), at chromosome 1 positions 1557353-1559122, 1559207-1560388 and 1560463-1561203 respectively (PhaC1 UniProt accession P23608; PhaAUniProt accession P14611; PhaB1 UniProt accession 14697). The locus-specific genes *phaC1* and *phaB1* are referred to hereafter as *phaC* and *phaB*, respectively. The three coding sequences were treated as separate open reading frames (ORF). Their coding boundaries, start codons, terminal stop codons and translated products were recorded before downstream construct design. Each coding sequence translated to a single ORF with no internal stop codon and full identity to its reference protein. The operon was selected because it represents one of the extensively characterised PHB biosynthetic systems and has been successfully expressed in a variety of microbial hosts, making it an appropriate model pathway for computational strain engineering studies [22,23]. PHB biosynthesis has also been characterised in taxonomically diverse producers more broadly [26]. The operon encodes the three core enzymes required for PHB biosynthesis: *PhaA* (acetyl-CoA acetyltransferase), *PhaB* (acetoacetyl-CoA reductase), and *PhaC* (PHB synthase). Sequence integrity, open reading frame preservation and translational continuity were assessed using Clustal Omega (v1.2.4) prior to downstream construct design. The genes were handled as three separate open reading frames throughout, and no fused *PhaCAB* product was generated. The bioinformatic tools and sequence resources used are summarised in Table 2.

### 2.2. In Silico Physicochemical Characterisation of phaCAB-Encoded Proteins

**Figure 2:**
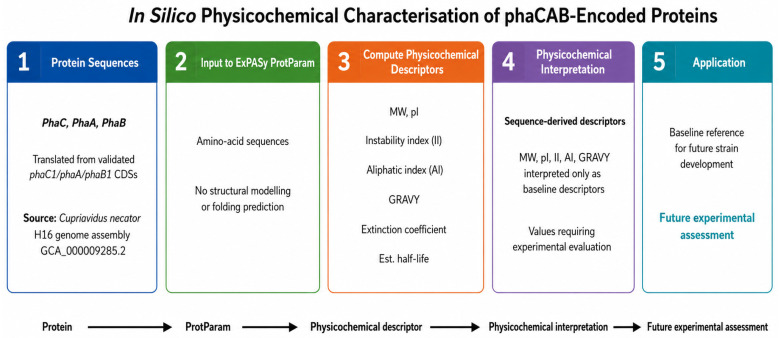
*In silico* physicochemical characterisation workflow for the phaCAB-encoded proteins. The validated *PhaC*, *PhaA* and *PhaB* amino-acid sequences from *C. necator* were analysed using ExPASy ProtParam to calculate molecular weight, theoretical isoelectric point, instability index, aliphatic index, GRAVY, extinction coefficient and estimated half-life. These outputs were interpreted only as baseline sequence-derived physicochemical descriptors and do not predict protein folding, solubility, catalytic activity, expression performance or host compatibility. The estimated half-life is an *E. coli* in vivo reference value and is not assumed to extrapolate to *T. reesei* or thermophilic *C. thermocellum*. The results provide reference information for future experimental assessment and strain development.

The physicochemical properties of *PhaA*, *PhaB* and *PhaC* were analysed using the ExPASy ProtParam platform, as shown in Figure 2. Parameters including molecular weight, theoretical isoelectric point, instability index, aliphatic index and hydropathicity were examined. These parameters were reported as baseline sequence-derived physicochemical descriptors and were not used to predict solubility, expression performance, protein folding, catalytic activity or host compatibility. No structural modelling or protein folding predictions were performed.

Physicochemical characterisation was included to provide baseline sequence-derived descriptors of the PHB biosynthetic enzymes and to identify any values that may warrant attention in future experimental implementation. These parameters were not used to predict solubility, expression performance, protein folding, catalytic activity, cofactor availability or host compatibility and are interpreted only as preliminary physicochemical descriptors.

### 2.3. Host-Specific Vector Selection and Expression Design

Expression vectors were selected according to the physiological and transcriptional characteristics of each host system. pET-24a(+) was selected as an intermediate cloning vector for *E. coli* BL21 (DE3). For *T. reesei*, three independent expression cassettes were designed, each with its own promoter and terminator rather than a single polycistronic or bidirectional-promoter arrangement, reflecting the limited characterisation of multi-gene expression architectures in this host [27], each combining the constitutive *tef1* promoter, a fungal Kozak context, one coding sequence and a *cbh1* terminator, a validated fungal selectable marker (*hph*) and 5′ and 3′ homology arms (approximately 1000 bp each) targeting a candidate integration region, designated NL1 (candidate site: *T. reesei* RUT C-30 assembly GCA_000513815.1, scaffold_6:142,300–144,300, + strand; coordinates given in half-open, 0-based format [start, end), consistent with standard genomic interval notation, so that the 5′ arm [142,300–143,300) and 3′ arm [143,300–144,300) are each exactly 1000 bp with no overlap, combining to exactly 2000 bp; the site is a computationally proposed candidate and its neutrality requires experimental confirmation), and is designed to preserve the native *cbh1* locus and its cellulolytic function, consistent with reports that integrating heterologous genes away from the native *cbh1* locus can avoid disrupting endogenous cellulase expression and, depending on the alternative site, may support higher heterologous transcription than *cbh1*-locus integration itself [28,29]. For *C. thermocellum*, pIKM1 was used as the shuttle backbone and supplemented with a host-compatible *groEL* promoter, individual ribosome-binding sites, appropriate intergenic spacing and a transcription terminator. Because promoter strength cannot be established conclusively from sequence analysis alone, promoter compatibility was assessed from documented function in the intended host. Quantitative promoter strength and condition-specific activity remain to be determined experimentally. Vector selection was based on host compatibility, promoter availability and suitability for heterologous expression studies reported in the literature [18,19].

### 2.4. Primer Design and Restriction Site Engineering

Primers were designed to support intermediate assembly in *E. coli* and host-specific construction for *T. reesei* and *C. thermocellum*. For the intermediate construct, BamHI and SalI sites flanked the assembled insert in pET-24a(+). For *C. thermocellum*, KflI and XbaI sites flanked the *groEL*-driven cassette for insertion into pIKM1. For *T. reesei*, specific gene primers carried fungal Kozak contexts and overlap tails for cassette assembly rather than a single directional insertion, so that each gene formed an independent cassette. Melting temperature, GC content and site incorporation were evaluated in SnapGene (v6.2) to confirm theoretical compatibility. The individual coding sequences were screened to confirm the absence of internal recognition sites that could interfere with assembly. The other key factors were vector compatibility, preservation of coding sequence integrity and maintenance of promoter-gene orientation required for heterologous expression.

### 2.5. Host-Specific Codon-Usage and Sequence-Composition Analysis

Because successful virtual assembly does not establish expression feasibility, a specific host compatibility analysis was performed for each coding sequence in the two final hosts and, for comparison, in *E. coli* BL21 (DE3), the intermediate assembly host. For *phaC*, *phaA* and *phaB* against both *T. reesei* and *C. thermocellum*, the following parameters were evaluated: gene length; coding-sequence GC content; host genome GC content and the difference between them; codon adaptation index (CAI); effective number of codons; rare-codon frequency and clustering.

The coding sequences of *phaC*, *phaA* and *phaB* were analysed separately after removal of the terminal stop codon for codon-based calculations. Host-specific codon-adaptation reference tables were generated from annotated ribosomal-protein coding sequences of the respective host assemblies. The *C. thermocellum* reference set comprised 56 ribosomal-protein coding sequences from assembly GCF_000184925.1, and the *T. reesei* reference set comprised 100 ribosomal-protein coding sequences from assembly GCA_000513815.1. In both cases, coding sequences annotated as ribosomal proteins in the respective RefSeq/GenBank annotation were selected, excluding pseudogenes and partial CDSs. For *E. coli*, the built-in Biopython SharpEcoliIndex reference set (Bio.SeqUtils. CodonUsageIndices), derived from the highly expressed *E. coli* gene set of Sharp and Li [30], was used, and genome GC content was calculated from assembly GCF_000009565.1. Native *C. necator* H16 coding sequences were retrieved from assembly GCA_000009285.2.

The codon adaptation index was calculated using the Sharp–Li relative-adaptiveness method [30] implemented in Biopython 1.86. Codons absent from a host reference set were assigned a pseudocount of 1 before normalisation. The effective number of codons was calculated according to the Wright method [31] as Nc = 2 + 9/F2 + 1/F3 + 5/F4 + 3/F6, where F values represent synonymous-family homozygosity.

Gene GC content was calculated from the complete coding sequence, including its terminal stop codon. Host GC content was calculated directly from unambiguous A, C, G and T bases in the corresponding genome assembly. GC difference was calculated as gene GC% minus host genome GC% and is reported in percentage points.

A sense codon was classified as rare when its specific host relative-adaptiveness value was below 0.10. Rare-codon frequency was calculated as the number of rare codons divided by the total number of sense codons in the stop-excluded coding sequence, multiplied by 100. A rare-codon cluster was defined as a 10-codon sliding window containing at least three rare codons. Overlapping qualifying windows were merged and reported as one cluster. Several methodological caveats apply to this analysis. CAI values were calculated against different host-specific reference sets (the built-in Biopython SharpEcoliIndex for *E. coli*; ribosomal-protein reference sets assembled for *T. reesei* and *C. thermocellum*) and are not a directly equivalent absolute scale across hosts; they should be read as within-host relative measures rather than a cross-host performance ranking. The pseudocount of 1 assigned to codons absent from a reference set was not varied, so its quantitative effect on the reported CAI values has not been evaluated. Whole-genome GC content is a coarse comparator, particularly for the eukaryotic *T. reesei* genome, where coding-region GC and third-codon-position (GC3) content would be more informative than a single genome-wide value; the GC comparisons reported here should be interpreted with this limitation in mind. The 0.10 relative-adaptiveness threshold used to classify rare codons, and the 10-codon/three-rare-codon window used to define clusters, follow common conventions in the codon-usage literature but were not subjected to a sensitivity analysis in this study; different thresholds could plausibly shift which codons and windows are flagged.

Ribosome-binding sites for the *C. thermocellum* construct were designed within the native *groL* (*groEL*) 5′ leader of *C. thermocellum* DSM 1313 (NC_017304.1, immediately upstream of position 486968). Predicted translation-initiation rates were calculated with OSTIR v1.1, an open-source implementation of the RBS Calculator thermodynamic model, using a host-specific anti-Shine–Dalgarno sequence (5′-ACCTCCTTT-3′) taken from the 3′ terminus of the *C. thermocellum* 16S ribosomal RNA (locus CLO1313_RS02235). Shine–Dalgarno cores and spacer lengths were varied systematically, and the first 99 nucleotides of each native coding sequence were supplied as the downstream context. Predicted initiation rates are reported in arbitrary units on the RBS Calculator scale and are relative rather than absolute measures; the underlying model is parameterised for *E. coli* at 37 degrees Celsius and has not been calibrated for thermophilic translation at 55 degrees Celsius.

### 2.6. Computational Construct Validation

Constructs were validated computationally with Clustal Omega (v1.2.4) and SnapGene (v6.2). Alignments verified preservation of each open reading frame after assembly. Simulated restriction digestion and virtual agarose-gel electrophoresis were used to check whether predicted fragment sizes matched the expected vector and insert architecture. These analyses provided construct-level computational consistency and did not demonstrate laboratory amplification, ligation efficiency, transformation or construct stability.

### 2.7. In Silico Cloning and Host-Specific Subcloning Strategy

**Figure 3:**
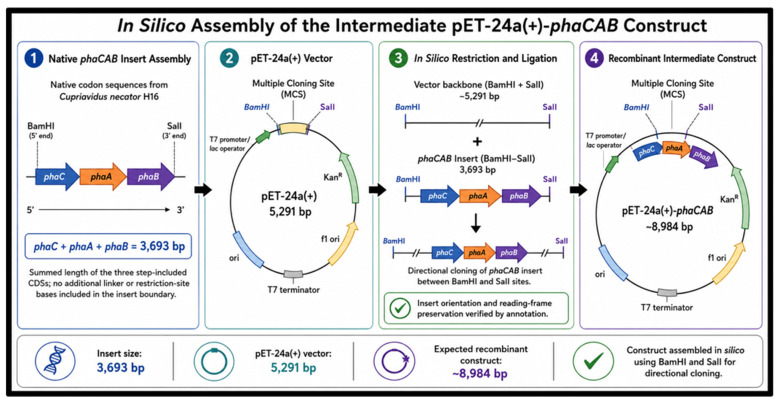
*In silico* assembly of the intermediate pET-24a(+)-phaCAB construct. The native *phaC*, *phaA* and *phaB* coding sequences were assembled as a 3693 bp insert (the summed length of the three stop-included CDSs, with no additional linker or restriction-site bases included in the insert boundary), flanked by BamHI and SalI sites, and ligated into the 5291 bp pET-24a(+) vector to generate an approximately 8984 bp recombinant construct.

For *T. reesei*, three *tef1*-driven cassettes were assembled *in silico*, each carrying a fungal Kozak context, one coding sequence and a *cbh1* terminator, an *hph* selection cassette and candidate NL1 homology arms (designed to preserve the native *cbh1* locus). For *C. thermocellum*, a single *groEL*-driven cassette containing individually tuned ribosome-binding sites upstream of *phaC*, *phaA* and *phaB* and a downstream terminator was assembled into pIKM1. Insert orientation and reading frame preservation were verified through annotation of the resulting construct maps. The overall cloning strategy was designed to evaluate the feasibility of integrating the PHB biosynthetic pathway into cellulolytic hosts with metabolic characteristics relevant to CBP. The initial phase was the 3693 bp *phaCAB* insert assembly into the 5291 bp pET-24a(+) vector to generate an approximately 8984 bp intermediate construct before development of the host-specific expression systems, as shown in Figure 3.

The *in silico* cloning workflow consisted of sequence amplification, restriction enzyme digestion, vector linearisation and ligation simulation using SnapGene’s construct assembly module (San Diego, CA, USA). Restriction digestion was simulated under default enzyme recognition parameters, and insert orientation was verified through annotation of open reading frames within the resulting plasmid maps.

### 2.8. In Silico Subcloning into T. reesei

**Figure 4:**
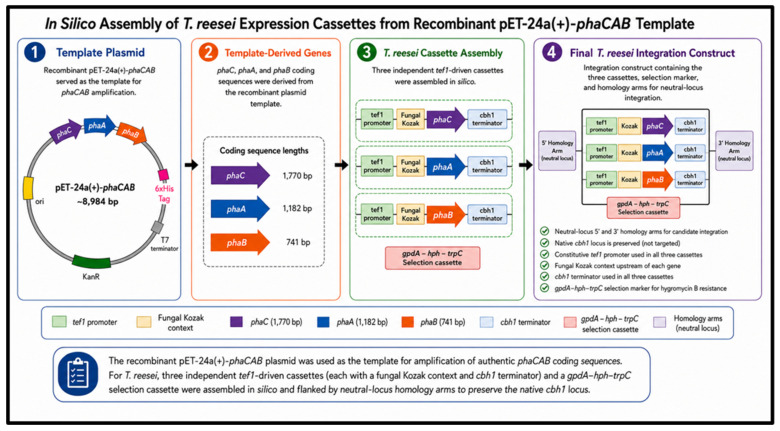
*In silico* assembly of the host-specific *T. reesei* expression construct (TrePHB3 integration construct). The native *phaC* (1770 bp), *phaA* (1182 bp) and *phaB* (741 bp) coding sequences were incorporated into three independent *tef1*-driven cassettes, each containing a fungal Kozak context and a *cbh1* terminator. The final construct also contained a *gpdA–hph–trpC* selection cassette and candidate NL1 5′ and 3′ homology arms designed to preserve the native *cbh1* locus.

The recombinant pET-24a(+)-*phaCAB* plasmid was used as the template for per-gene amplification into three cassettes. For *T. reesei*, three *tef1*-driven expression cassettes were assembled *in silico*, each comprising a fungal Kozak sequence, a single coding sequence and a *cbh1* terminator. The final construct also contained an *hph* selection cassette and candidate NL1 homology arms as illustrated in Figure 4.

### 2.9. In Silico Subcloning into C. thermocellum

**Figure 5:**
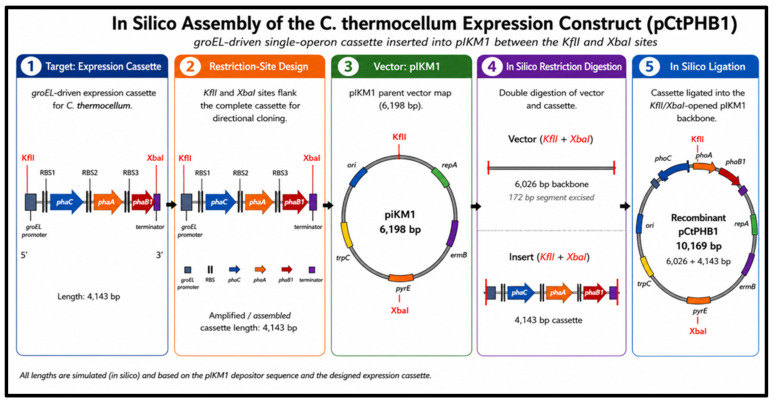
*In silico* assembly of the *C. thermocellum* expression construct (pCtPHB1). The *groEL*–RBS1-*phaC*–RBS2-phaA–RBS3-phaB–terminator expression cassette (4143 bp) was assembled *in silico* and inserted between the KflI and XbaI sites of the 6198 bp pIKM1 backbone (Addgene depositor sequence), which retains 6026 bp after excision of a 172 bp native segment between the two sites, giving a 10,169 bp final construct.

The recombinant pET-24a(+)-*phaCAB* construct was used as the source template for simulated subcloning into the pIKM1 vector. The three coding sequences were used as the basis for a complete expression cassette comprising the *groEL* promoter (200 bp), RBS1–*phaC* (35 bp ribosome-binding site plus the 1770 bp *phaC* coding sequence), a 40 bp intergenic region, RBS2–*phaA* (35 bp ribosome-binding site plus the 1182 bp *phaA* coding sequence), a second 40 bp intergenic region, RBS3–*phaB* (35 bp ribosome-binding site plus the 741 bp *phaB* coding sequence), and a downstream 65 bp rho-independent terminator, giving a 4143 bp cassette that was assembled *in silico* and inserted between the KflI and XbaI sites of pIKM1, followed by virtual digestion of both insert and vector within SnapGene. Recombinant construct assembly was performed through simulated ligation, and sequence integrity was verified using Clustal Omega. The resulting construct was evaluated for theoretical compatibility with the thermophilic host *C. thermocellum*, as shown in Figure 5.

*In silico* pathway design is increasingly used in metabolic engineering to reduce experimental costs and identify feasible cloning strategies before laboratory implementation. The computational framework developed in this study therefore provides a rational design step for future experimental strain construction.

### 2.10. Study Scope and Computational Limitations

This study was designed as a computational construct-development and feasibility assessment. No laboratory cloning, transformation, expression analysis or PHB production was conducted. Promoter activity, plasmid stability, transformation efficiency, transcriptional regulation, protein expression, metabolic flux and polymer accumulation were not experimentally evaluated. The constructs therefore represent candidate designs intended to guide future laboratory implementation and strain development.

## 3. Results and Discussion

### 3.1. Confirmation of phaCAB Sequence Retrieval and Translation

**Figure A1:**
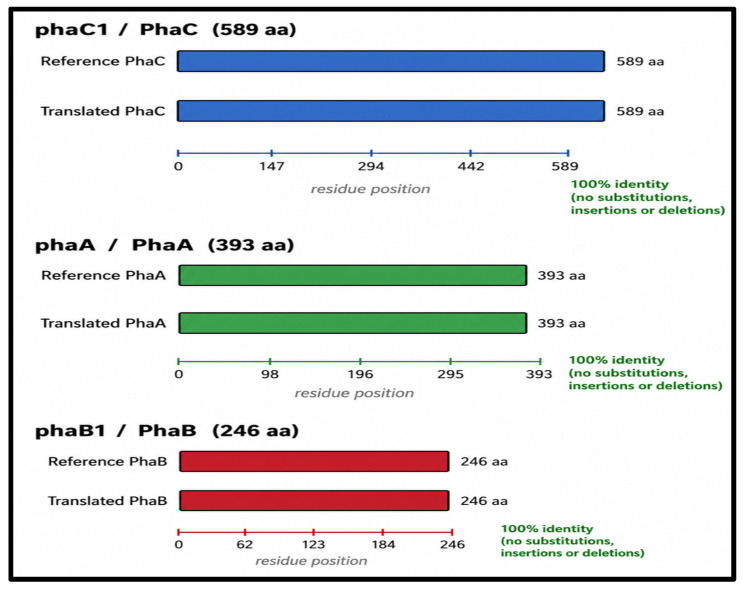
Schematic protein-level identity summary for the retrieved *phaC1*, *phaA* and *phaB1* coding sequences: three separate schematic reference-versus-translated identity checks, each analysed as an independent open reading frame.

The retrieved native *phaC1*, *phaA* and *phaB1* coding sequences from *C. necator* (assembly GCA_000009285.2) were translated individually and aligned with their respective reference proteins using Clustal Omega. Summarised schematically in Figure A1, we observed 100% identity between each translated sequence and its corresponding reference protein, confirming preservation of the open reading frames (ORFs) and the absence of substitutions, insertions, or deletions throughout the translation workflow. These results indicate that the coding regions remained intact and suitable for downstream cloning and construct development.

The 100% identity here reflects faithful retrieval and translation of the deposited *C. necator* H16 coding sequences, not cross-species conservation.

The confirmed sequence integrity provided confidence for subsequent physicochemical characterisation, primer design, and *in silico* cloning analyses. The 100% identity is a quality-control check confirming the accuracy of the sequence-retrieval and translation workflow used in this study; it is not itself a substantive biological finding.

### 3.2. Physicochemical Characterisation of the phaCAB-Encoded Proteins

#### 3.2.1. PhaA (Acetyl-CoA Acetyltransferase)

The *phaA* gene encodes the acetyl-CoA acetyltransferase protein, characterised in Table 3.

In several bacteria, *phaA* is clustered with *phaB* (acetoacetyl-CoA reductase) in an operon, suggesting coordinated expression during PHB production. However, in some species, a transcriptional stop signal between *phaA* and *phaB* may uncouple their expression. *PhaA* is responsible for the initial condensation of two acetyl-CoA molecules to form acetoacetyl-CoA, thereby initiating PHB biosynthesis [32]. The enzyme consists of 393 amino acids [32], with a calculated molecular weight of 40.87 kDa and a theoretical isoelectric point (pI) of 5.85, indicating a mildly acidic character. The instability index (28.9) classifies the enzyme as stable. The aliphatic index was comparatively high at 92.4; however, actual thermal stability requires experimental testing. The negative GRAVY value (−0.12) indicates overall hydrophilic character. Although computational predictions cannot confirm expression success, these characteristics provide preliminary support for future experimental expression studies.

#### 3.2.2. PhaB (Acetoacetyl-CoA Reductase)

*PhaB*, also known as acetoacetyl-CoA reductase, is a key enzyme in the PHA biosynthetic pathway and is described in Table 4. It catalyses the NADPH-dependent reduction of acetoacetyl-CoA to (R)-3-hydroxybutyryl-CoA, which is subsequently polymerised by PHA synthase to form PHAs such as polyhydroxybutyrate (PHB).

The enzyme is comparatively smaller, with ≈246 amino acids, a molecular weight of 26.45 kDa and a near-neutral theoretical pI of 6.32. The instability index (31.7) predicts stable protein, and the aliphatic index (84.6) is comparatively high; actual thermal stability requires experimental testing. The slightly negative GRAVY value (−0.05) indicates overall hydrophilic character; ProtParam does not predict subcellular localisation, and any co-factor interaction requires separate experimental or structural evidence.

#### 3.2.3. PhaC (Polyhydroxybutyrate Synthase)

*PhaC* is the key polymerase responsible for PHB chain elongation. The enzyme comprises approximately 589 amino acids with a molecular weight of 63.98 kDa [33], as characterised in Table 5. The instability index (35.4) remains below the threshold for instability, while the aliphatic index (88.1) is comparatively high; actual thermal stability requires experimental testing. The extinction coefficient reflects the abundance of aromatic residues (Trp, Tyr, Cys) and is principally useful for estimating protein concentration; it does not itself establish structural stability. The GRAVY value (−0.09) indicates overall hydrophilic character; ProtParam does not predict subcellular or granule-associated localisation.

Collectively, the ProtParam analyses indicate baseline sequence-derived physicochemical descriptors consistent with stable, hydrophilic proteins; actual expression, folding, activity and host compatibility require experimental validation.

Similar physicochemical characteristics have been reported for PHB biosynthetic enzymes from *C. necator*, which are typically stable intracellular proteins involved in polymer synthesis pathways. These sequence-derived physicochemical descriptors provide baseline information for future heterologous-expression studies but do not predict host compatibility or expression performance.

Comparable ProtParam-based physicochemical profiling of PHA synthase (*PhaC*) enzymes across other industrially relevant PHA-producing genera (*Azotobacter*, *Bacillus*, *Cupriavidus* and *Halomonas*) reported instability indices for *Cupriavidus PhaC* homologues of approximately 34–43 and negative GRAVY values between −0.27 and −0.16, broadly consistent with the stable, hydrophilic character (instability index 35.4, GRAVY −0.09) obtained here for *PhaC* from *C. necator* H16 [34]. This agreement across independent *in silico* analyses of different *PhaC* sequences supports the general reliability of ProtParam-derived descriptors as a preliminary screening step; however, as in that study, such sequence-derived predictions alone cannot substitute for experimentally resolved structural or functional data, particularly given the reported structural divergence of Bacillus-derived PHA synthases from the otherwise structurally conserved Azotobacter, Cupriavidus and Halomonas group [34].

### 3.3. Host-Specific Codon and Regulatory Compatibility

The native *phaC*, *phaA* and *phaB* coding sequences from *C. necator* H16 displayed different codon-usage profiles across the two final hosts and the *E. coli* intermediate comparator. For *E. coli*, CAI values were 0.7666, 0.7695 and 0.7544 for *phaC*, *phaA* and *phaB* respectively, and no codon in any of the three genes fell below the rare-codon threshold. *E. coli* showed the highest codon compatibility, although it was included only as an intermediate comparator.

The lowest CAI values were obtained for *C. thermocellum*, at 0.4832 for *phaC*, 0.4912 for *phaA* and 0.4898 for *phaB*. This host also showed the largest compositional mismatch, with the GC contents of *phaC*, *phaA* and *phaB* exceeding the host genome GC content by 27.69, 29.04 and 24.01 percentage points respectively. Rare-codon frequencies were 5.26% for *phaC*, 3.05% for *phaA* and 4.07% for *phaB*, and the dominant host-disfavoured codon in all three genes was the arginine codon CGC, which is strongly avoided in this AT-rich thermophile. Two rare-codon clusters were identified in *phaC* and one in *phaB*.

Intermediate-to-high CAI values were obtained for *T. reesei*, at 0.6582 for *phaC*, 0.7356 for *phaA* and 0.7111 for *phaB*. The compositional mismatch was correspondingly smaller, at 13.50, 14.85 and 9.82 percentage points. Rare codons were scarce, at 0.68% for *phaC* and 0.51% for *phaA*, with none detected in *phaB*, and no rare-codon cluster was identified in any of the three genes. The principal host-disfavoured codons were AAA and GTA.

ENC values were 31.17 for *phaC*, 27.76 for *phaA* and 29.28 for *phaB*. These values indicate moderately biased synonymous-codon usage, consistent with the high GC content of the *C. necator* genome (66.34%) and typical of native bacterial coding sequences.

**Figure 6:**
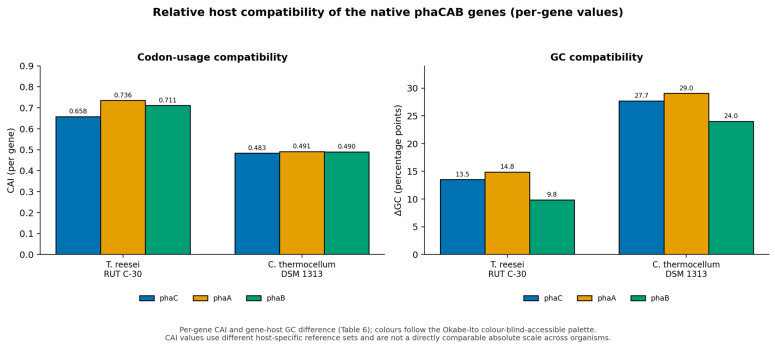
Relative host compatibility of the native *phaCAB* genes, shown as numerical values rather than qualitative categories. Codon-usage compatibility is given as the mean CAI of *phaC*, *phaA* and *phaB*, and GC compatibility as the mean absolute gene–host GC difference: mean CAI 0.702 and mean |ΔGC| 12.72 percentage points for *T. reesei*, and mean CAI 0.488 and mean |ΔGC| 26.91 percentage points for *C. thermocellum*. Exact gene-specific values are reported in Table 6. CAI values were computed against different host-specific reference sets and are not a directly comparable absolute performance scale across organisms; they should be read as within-host relative measures only.

This is reflected in the relative codon-usage compatibility and rare-codon load (Table 6 and Figure 6), which diverge moderately from *T. reesei* and strongly from *C. thermocellum*, so codon optimisation is more critical for the thermophile. The pCtPHB1 construct reported here deliberately retains the native *C. necator* coding sequences as an unoptimised reference design: it establishes a baseline against which the magnitude of the predicted codon-usage mismatch can be judged, and avoids introducing untested synonymous substitutions ahead of any experimental data on their effect. A codon-optimised pCtPHB1 alternative, incorporating the *C. thermocellum*-preferred codons identified here (in particular replacement of the rare CGC arginine codon and disruption of the identified rare-codon clusters), is a logical next design iteration but was not generated computationally in this study; the native-CDS construct should therefore be read as a reference point rather than a final expression-ready design for this host.

The genes showed moderately high CAI values relative to the selected *E. coli* reference set, intermediate-to-high values in *T. reesei* and the lowest values in *C. thermocellum*. The largest compositional mismatch occurred in *C. thermocellum*, where the genes were 24.01–29.04 percentage points more GC-rich than the host genome. In *T. reesei*, the GC differences were 9.82–14.85 percentage points, rare codons were scarce and no rare-codon clusters were detected, although isolated host-disfavoured AAA and GTA codons were identified.

The ENC values of approximately 28 to 31 indicate moderate synonymous-codon bias. This is consistent with the high-GC genome of *C. necator* H16 and confirms that native coding sequences, rather than an engineered deposit, underpin the compatibility analysis reported here.

Positive values indicate that the gene is more GC rich than the host genome, whereas negative values indicate that it is less GC rich. Here, ΔGC was calculated as

ΔGC = GC(gene) − GC(host)

The principal host-disfavoured codons in the *T. reesei* comparison were AAA and GTA, based on the defined relative-adaptiveness threshold of 0.10.

Rare-codon analysis reinforced this pattern (Table 7). In the *C. thermocellum* comparison, 3.05 to 5.26% of sense codons fell below the relative-adaptiveness threshold, predominantly the arginine codon CGC, and the genes were 24.01 to 29.04 percentage points more GC rich than the host genome, with two clusters in *phaC* and one in *phaB*. In contrast, *T. reesei* showed rare-codon frequencies of 0 to 0.68% and no clusters. The results do not support a single universal optimisation strategy. The thermophilic bacterial construct requires attention to the substantial GC mismatch, the CGC arginine load and host-specific ribosome-binding-site architecture, whereas the fungal construct requires comparatively minor synonymous adjustment. Experimental expression data will ultimately be required to determine whether further recoding is necessary.

This host-dependent divergence in codon compatibility parallels findings from a comparative evaluation of open-source codon harmonisation tools across *Escherichia coli*, Saccharomyces cerevisiae and Streptomyces lividans, which found that no single harmonisation strategy performed uniformly well across hosts and that gene-specific features such as GC content and RNA secondary structure significantly influenced outcomes [35]. That study also cautioned that indiscriminate removal of rare codons can eliminate functionally important rare-codon clusters that assist co-translational folding, rather than simply removing translational bottlenecks [35]. This reinforces the conservative choice made here to retain the native *C. necator* coding sequences for pCtPHB1 as an unoptimised reference construct rather than pre-emptively substituting the identified rare-codon clusters in phaC and phaB without experimental evidence of their functional role.

### 3.4. Assembly of the Intermediate E. coli Construct

**Figure 7:**
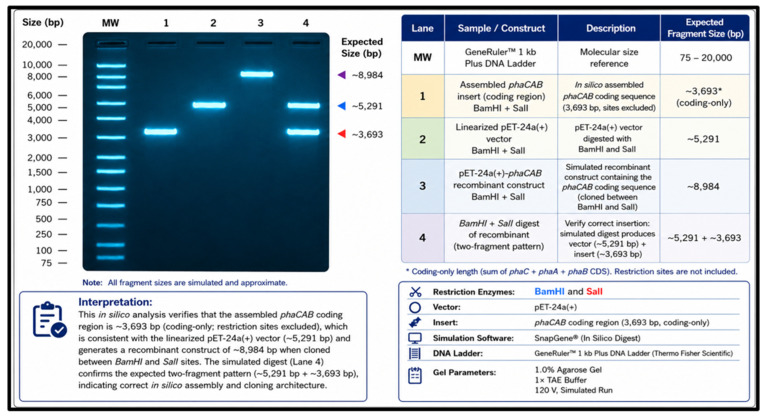
Simulated agarose-gel validation of the intermediate pET-24a(+)-phaCAB construct. Lane MW: molecular size ladder. Lane 1: coding-only *phaCAB* insert (approximately 3693 bp; summed stop-included CDS length, excluding primer-added restriction-site bases). Lane 2: linearised pET-24a(+) vector backbone (approximately 5291 bp). Lane 3: intact recombinant pET-24a(+)-*phaCAB* construct (approximately 8984 bp). Lane 4: BamHI/SalI digest of the recombinant construct, releasing the linearised vector backbone and the *phaCAB* insert as the expected two-fragment pattern.

The individual genes were assembled into pET-24a(+) as a convenient intermediate carrier. Simulated restriction digestion and virtual agarose-gel electrophoresis produced fragment sizes consistent with the expected architecture (Figure 7): the coding-only insert at approximately 3693 bp, the linearised vector at approximately 5291 bp and the recombinant construct at approximately 8984 bp, with the expected two-fragment pattern on BamHI and SalI digestion. This simulated digest serves as a design-verification (quality-control) check confirming that the construct sequence is internally consistent with its intended architecture; it does not constitute experimental confirmation of amplification, ligation efficiency, gene expression or PHB production, and the pET-24a(+) construct does not establish expression in either final host. The pET-24a(+) intermediate is used here for convenience rather than scientific necessity: because the three coding sequences were first assembled and computationally sequence-checked as a single annotated unit in a well-characterised bacterial backbone, this step provided a common, easily inspected reference sequence from which the two host-specific constructs (TrePHB3 integration construct and pCtPHB1) could each be derived computationally, rather than assembling each host-specific design independently from the three separate source CDSs. The intermediate is retained only as an assembly and template-supply step.

From a circular bioeconomy perspective, the *in silico* preservation of the intended pathway architecture represents the first step toward developing microbial systems capable of converting lignocellulosic residues such as HH into biodegradable polymers. Although no experimental expression studies were conducted, the construct architecture provides a theoretical framework for future strain development and pathway implementation.

### 3.5. Host-Specific T. reesei Construct Design (TrePHB3 Integration Construct)

A dedicated fungal expression architecture was designed for *T. reesei*, suited to filamentous-fungal expression. Three independent cassettes were assembled, each combining the constitutive *tef1* promoter, a fungal Kozak context, one coding sequence and a *cbh1* terminator (Table 8).

Promoter compatibility was assessed from the documented function of the *tef1* promoter and *cbh1* terminator in *T. reesei*; quantitative promoter strength remains to be determined experimentally. Selection is provided by a validated *gpdA–hph–trpC* cassette. Integration is directed by candidate NL1 5′ and 3′ homology arms designed to preserve the native *cbh1* locus and its cellulolytic function. Ectopic integration under hygromycin selection remains a possible alternative. The assembled construct, designated TrePHB3 integration construct, contains three independent expression cassettes of approximately 2620, 2032 and 1591 bp, an approximately 2100 bp selection cassette and approximately 2000 bp of combined homology arms, giving a fully assembled size of 10,343 bp; this total is the exact arithmetic sum of the stated component design values rather than an independently verified sequence length, since the regulatory-part lengths (promoter, Kozak context, terminator, homology arms) are representative design values rather than fixed experimentally determined sequences. This design accounts for fungal gene-expression architecture but does not demonstrate transcription, intracellular PHB accumulation or the effect of the added biosynthetic burden on cellulase secretion and carbon partitioning, all of which require experimental testing. TrePHB3 integration construct is designed as a named collection of three assembled *tef1*-Kozak-CDS-*cbh1* integration cassettes plus the gpdA-hph-trpC selection cassette and NL1 homology arms, intended for genomic integration at the candidate NL1 site rather than as an autonomously replicating circular plasmid; no fungal replication origin or plasmid backbone is included in the reported 10,343 bp total, and the “p” designation refers to the assembled DNA construct rather than a self-replicating vector. Repeating the same *tef1* promoter and *cbh1* terminator sequence in all three cassettes introduces sequence redundancy that could in principle increase the risk of intramolecular recombination, cassette rearrangement or transcriptional interference between adjacent modules; this risk was not assessed computationally in this study. Diversifying the promoter and terminator sequences used across the three cassettes, for example by substituting alternative constitutive *T. reesei* promoters and terminators of comparable strength, would be a reasonable design modification to reduce this redundancy and is noted here as a direction for future design iterations rather than a change implemented in the present construct.

**Figure 8:**
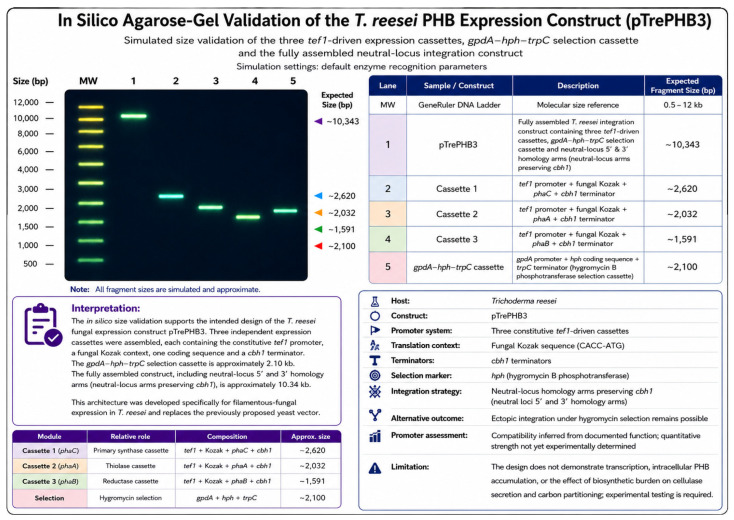
*In silico* agarose-gel size validation of the *T. reesei* TrePHB3 integration construct. Lane 1 represents the intact approximately 10,343 bp integration construct; lanes 2–4 represent the three independent *tef1*-driven expression cassettes carrying *phaC*, *phaA* and *phaB* at approximately 2620, 2032 and 1591 bp, respectively; and lane 5 represents the approximately 2100 bp *gpdA–hph–trpC* selection cassette.

The individual design choices underlying this architecture can also be weighed against the literature rather than taken only on documented function. The gpdA–hph–trpC selection cassette used here originates from a heterologous *Aspergillus nidulans* expression system [36]; a *T. reesei*-homologous alternative built from the native pki1 promoter and cbh1 terminator has been reported to give a 15- to 20-fold higher transformation frequency than this heterologous gpdA/trpC-type cassette [37], so the selection strategy adopted here may be more conservative than necessary for this host. The choice of a candidate NL1 site over the *cbh1* locus is similarly a design decision rather than a validated one: *cbh1* remains the only integration site in *T. reesei* with a documented positive effect on heterologous gene expression, and NL1 has not, to our knowledge, been independently benchmarked against it. A further consideration follows from the sequence redundancy already noted above: a *tef1*-promoter/*cbh1*-terminator cassette on a circular plasmid has been shown to integrate preferentially by homologous recombination immediately upstream of the native *tef1* locus rather than at the intended site, driven by sequence identity with the promoter itself [18]. Because the TrePHB3 design places three copies of the same *tef1* promoter in a single construct, this precedent raises the possibility that integration could be redirected toward the native *tef1* locus, or toward one of the construct’s own internal promoter copies, rather than being confined to the intended NL1 site; this risk was not evaluated computationally here and would need to be assessed experimentally, for example by junction PCR and sequencing at both the intended locus and the native tef1 locus (Figure 8).

The *tef1*/*cbh1* cassette architecture adopted for the TrePHB3 construct reflects a previously validated expression strategy in *T. reesei*, in which the *tef1* promoter has repeatedly been used to drive constitutive heterologous expression. However, a recent review of *T. reesei* as a heterologous protein production host emphasises that yields of non-native proteins in this organism remain frequently limited by proteolytic degradation and by incompatibility between the fungus’s secretory machinery, which is highly optimised for native cellulases, and heterologous protein cargo, with multi-protease gene deletion and signal-peptide screening identified as necessary complementary strategies rather than promoter choice alone [38]. This suggests that the present computational design, while establishing a plausible transcriptional architecture, addresses only one of several bottlenecks likely to determine actual *PhaC*, *PhaA* and *PhaB* expression levels in *T. reesei*, and that protease-deficient or secretion-engineered chassis strains may need to be considered alongside this construct in future experimental work.

### 3.6. Host-Specific C. thermocellum Construct Design

For *C. thermocellum*, pIKM1 was retained as the shuttle backbone but supplemented with a complete candidate expression cassette. The cassette places the *groEL* promoter upstream of a *phaC*, *phaA*, *phaB* arrangement in which each gene is preceded by its own ribosome-binding site, with a rho-independent terminator downstream. The three ribosome-binding sites were designed with a graded predicted-strength hierarchy (Table 9) as a design hypothesis to be tested experimentally, not as a quantitatively reliable expression prediction for *C. thermocellum*. The highest predicted initiation rate (approximately 40,700 arbitrary units) was assigned to the site preceding *phaB* rather than *phaC*, on the hypothesis that rapid consumption of the acetoacetyl-CoA generated by *PhaA* (via the NADPH-dependent *PhaB* reductase step) would limit accumulation of this intermediate; an intermediate predicted rate (approximately 10,200 arbitrary units) was assigned to *phaA*, and a lower predicted rate (approximately 3300 arbitrary units) to *phaC*, giving an approximate order-of-magnitude predicted range across the cassette. These OSTIR outputs were not tested against alternative RBS sequences, spacer lengths or downstream sequence contexts, so the robustness of this ranking to such changes is unknown; the values should not be read with more precision than a coarse low/intermediate/high ordering, and predicted initiation rate is distinguished here from actual protein abundance or pathway flux, neither of which was measured.

**Figure 9:**
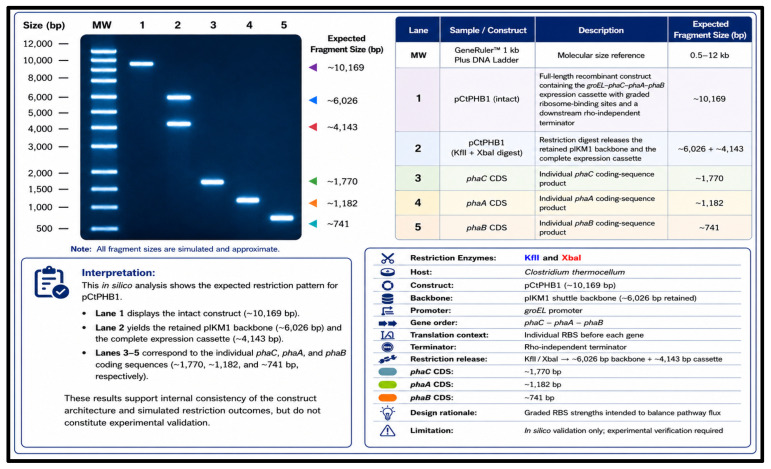
*In silico* agarose-gel validation of the *C. thermocellum* pCtPHB1 construct. Lane 1 represents the intact 10,169 bp construct. Lane 2 represents the KflI/XbaI digest, yielding the 6026 bp retained pIKM1 backbone and the 4143 bp complete *groEL–phaC–phaA–phaB–terminator* expression cassette. Lanes 3–5 represent the individual *phaC*, *phaA* and *phaB* coding-sequence products at 1770, 1182 and 741 bp, respectively.

Because *PhaA* produces acetoacetyl-CoA and *PhaB* consumes it in an NADPH-dependent reaction, the highest predicted initiation rate was assigned to *phaB*, an intermediate rate to *phaA* and a moderate rate to *phaC*, a design intended to test whether comparatively higher predicted *PhaB* initiation could reduce acetoacetyl-CoA accumulation rather than allowing it to build up. None of the designed Shine–Dalgarno or spacer sequences introduced a recognition site for any restriction enzyme used in the assembly workflow. These values are thermodynamic predictions for a mesophilic model and require experimental measurement in *C. thermocellum* before the intended translational hierarchy can be considered established. Simulated digestion of the resulting construct, designated pCtPHB1, returned fragment sizes consistent with the intended architecture (Figure 9).

The RBS spacer lengths designed here (5, 7 and 9 nt for phaC, phaB and phaA respectively) can be compared against an experimentally derived optimum for *C. thermocellum* gene expression. Using a chromosomally integrated reporter library, a 6–7 nucleotide gap between the ribosome-binding site and the start codon was identified as optimal for high expression in this organism [39]. Two of the three designed spacers in pCtPHB1 (phaC at 5 nt and phaA at 9 nt) fall outside this empirically optimal window, while the phaB spacer (7 nt) sits within it; combined with the fact that *phaB* was assigned the highest predicted initiation rate in the present design, this raises the possibility that the predicted low-to-intermediate initiation rates for *phaC* and *phaA* are partly an artefact of spacer geometry rather than solely of Shine–Dalgarno sequence strength, and spacer length should therefore be revisited experimentally alongside RBS sequence in any future optimisation of this construct.

### 3.7. Comparative Biological Feasibility of the Two Hosts

The two hosts present contrasting engineering profiles. *T. reesei* is an aerobic mesophile with a pentose phosphate pathway that can supply NADPH, an established genetic toolkit and a strong secretory system, but it diverts substantial carbon to cellulase secretion and directs acetyl-CoA into the tricarboxylic acid cycle and lipid synthesis. *C. thermocellum* is a thermophilic anaerobe with an exceptionally efficient cellulosome that degrades crystalline cellulose directly, which could remove the need for externally supplied enzymes, but its redox metabolism is dominated by NADH-linked fermentation, its acetyl-CoA is committed to ethanol, acetate, lactate and hydrogen formation, and its genetic accessibility is more limited. Temperature, oxygen requirement, cellulose-degradation strategy, acetyl-CoA and NADPH supply, gene-expression architecture, intracellular PHB burden and expected scale-up challenges therefore differ substantially between the two hosts, and each construct was designed to reflect these differences. Neither design has been tested, so the comparison indicates where experimental effort should focus rather than which host will perform better.

### 3.8. Metabolic and Physiological Constraints

The computational design and validation of *phaCAB* constructs in cellulolytic hosts provide a theoretical framework for future experimental evaluation of direct conversion of HH-derived carbohydrates into PHB through consolidated bioprocessing. By integrating lignocellulose degradation and polymer biosynthesis within a single microbial platform, the proposed strategy may reduce processing complexity, improve resource utilisation and support circular bioeconomy objectives through the valorisation of agricultural residues into biodegradable plastics.

#### 3.8.1. Acetyl-CoA Competition

Acetyl-CoA is a central metabolic intermediate required for multiple essential cellular processes, including energy generation, lipid biosynthesis, and fermentative metabolism. Introduction of the *phaCAB* pathway genes into cellulolytic hosts may therefore create competition between native metabolic pathways and PHB biosynthesis. In *C. thermocellum*, acetyl-CoA is naturally directed toward the production of ethanol, acetate, lactate, and hydrogen during anaerobic fermentation. Redirecting this metabolite toward PHB synthesis through the action of *PhaA* may reduce carbon availability for native energy-generating pathways and potentially affect cellular growth and substrate utilisation efficiency. Similarly, in *T. reesei*, acetyl-CoA is closely associated with the tricarboxylic acid (TCA) cycle, fatty acid biosynthesis, and cellular maintenance processes. Diverting acetyl-CoA toward PHB accumulation may alter intracellular carbon partitioning and influence fungal growth dynamics, cellulase secretion, and biomass formation. Consequently, balanced metabolic engineering strategies would likely be required to minimise pathway competition while maintaining efficient lignocellulose degradation.

This anticipated competition is consistent with broader experience in PHA metabolic engineering: a recent review of gene-deletion strategies for enhanced PHA production highlights that removal of competing pathways diverting acetyl-CoA or its precursors away from PHA biosynthesis is among the most consistently effective interventions for increasing PHA titre across diverse bacterial hosts [40]. This suggests that, beyond the *phaCAB* expression constructs designed here, deletion or downregulation of native fermentative or lipogenic pathways competing for acetyl-CoA in *C. thermocellum* and *T. reesei* respectively may ultimately be necessary to realise substantial PHB accumulation, rather than heterologous pathway expression alone.

#### 3.8.2. NADPH Availability

The reduction of acetoacetyl-CoA to (R)-3-hydroxybutyryl-CoA by *PhaB* requires NADPH as a reducing cofactor. The availability of NADPH is therefore a critical determinant of efficient PHB biosynthesis. In *C. thermocellum*, cellular redox metabolism is predominantly associated with NADH-dependent fermentative pathways, which may limit intracellular NADPH availability for PhaB activity. Insufficient NADPH regeneration could therefore reduce theoretical PHB biosynthetic efficiency and create metabolic imbalances within the engineered host. In contrast, *T. reesei* possesses active pentose phosphate pathway metabolism, which may provide a more favourable intracellular NADPH supply. However, NADPH is also required for amino-acid synthesis, oxidative stress responses, and lipid metabolism, potentially resulting in competition between native anabolic processes and PHB synthesis. Future metabolic engineering approaches may therefore require cofactor balancing strategies or enhancement of NADPH-generating pathways to improve theoretical PHB production efficiency.

#### 3.8.3. Host-Specific Expression Limitations

Although the computational design workflow demonstrated theoretical construct assembly feasibility, several host-specific biological constraints may influence successful heterologous expression of the *phaCAB* expression constructs. Codon usage differences between *C. necator* and the proposed expression hosts may affect translation efficiency and protein folding. In addition, promoter compatibility, plasmid maintenance, transcriptional regulation, mRNA stability and ribosome binding efficiency may vary between bacterial and fungal systems. The thermophilic anaerobic conditions required for *C. thermocellum* growth may also influence protein stability and folding of the heterologously expressed enzymes. In *T. reesei*, secretion-associated metabolic burden and intracellular compartmentalisation may further affect PHB biosynthesis. Since codon optimisation and transcriptional modelling were not performed in this study, the present work should be interpreted as a construct-design feasibility assessment rather than confirmation of functional gene expression.

#### 3.8.4. PHB Granule Formation Burden

Intracellular accumulation of PHB granules may impose physiological stress on engineered microbial hosts. Excessive polymer accumulation can alter cytoplasmic organisation, reduce metabolic flexibility, and increase cellular energy demand. In *C. thermocellum*, which naturally allocates substantial metabolic resources toward cellulolytic enzyme production and anaerobic fermentation, additional PHB biosynthesis may increase metabolic burden and reduce substrate conversion efficiency. Similarly, *T. reesei* dedicates significant cellular resources to extracellular cellulase secretion, and PHB accumulation may interfere with normal carbon utilisation and protein secretion pathways. The formation of intracellular polymer granules could therefore affect growth rates, enzyme secretion capacity, and overall biomass conversion performance. These potential physiological effects highlight the importance of balanced pathway regulation in future strain-engineering studies.

### 3.9. Proposed Experimental Validation

Experimental validation is required to confirm biological functionality. A proposed validation workflow is presented (Table 10), beginning with physical assembly or synthesis of the designed constructs and verification of complete sequence, orientation and junctions by restriction analysis and whole-plasmid sequencing. The host-specific constructs would then be transformed into *T. reesei* and *C. thermocellum*, and genomic integration or plasmid retention confirmed by junction PCR, sequencing and copy-number analysis. Transcription of *phaC*, *phaA* and *phaB* would be measured by RT-qPCR, and PhaC, PhaA and PhaB expression and activity assessed by Western blotting, targeted proteomics or enzyme assays. PHB would be screened qualitatively by Nile red or BODIPY staining and then quantified by gas chromatography with flame-ionisation detection or mass spectrometry after methanolysis, supported by Fourier-transform infrared spectroscopy as a supporting identification method rather than the sole quantitative approach. Construct stability would be evaluated by serial passaging with and without selection, and process performance assessed through sugar consumption, titre, yield and productivity, first on model sugars, then on HH hydrolysates and finally on pretreated HH solids. Appropriate controls include empty-vector strains, untransformed hosts, a recognised PHB-producing positive control and defined glucose or mixed-sugar media.

### 3.10. Biotechnology Implications of Heterologous phaCAB Expression in Cellulolytic Hosts

The computational incorporation of the *phaCAB* pathway genes into cellulolytic hosts represents only the first step towards the development of CBP for PHB production. For effective PHB biosynthesis to occur, the host organism must possess metabolic pathways capable of generating the precursor metabolites and reducing equivalents required by the heterologous pathway [41].

The *phaCAB* operon encodes three enzymes that convert acetyl-CoA into PHB through sequential condensation, reduction and polymerisation reactions [22,23], drawing on the acetyl-CoA and NADPH pools discussed in Section 3.8.1 and Section 3.8.2 above. Both *T. reesei* and *C. thermocellum* generate these metabolites through central carbon metabolism during growth on LB-derived carbohydrates [42,43], but whether this supply is sufficient to support heterologous PHB biosynthesis without constraining native growth remains to be experimentally determined [44].

Despite the theoretical compatibility of the pathway, several biological challenges remain. These include codon usage differences between donor and host organisms, transcriptional regulation, promoter strength, metabolic competition for acetyl-CoA, intracellular polymer accumulation and potential effects on host growth. Consequently, the present study should be viewed as a computational assessment of construct feasibility rather than confirmation of PHB production capability.

Nevertheless, the results suggest that integration of the *phaCAB* pathway genes into cellulolytic hosts provides a plausible route for coupling LB degradation with biodegradable polymer biosynthesis. Such an approach may support the future development of engineered CBP systems capable of converting agricultural residues such as HH into value-added biopolymers within an integrated biorefinery framework.

This single-organism strategy differs from an alternative CBP-PHA approach already demonstrated for LB, in which a highly cellulolytic but non-PHA-producing bacterium was co-cultured with a PHA-accumulating partner strain, together converting untreated Miscanthus biomass directly to PHB without any heterologous pathway engineering in either organism, achieving approximately 40 mg PHB per gram of biomass [45]. That co-culture result shows that division of labour between a degrader and a producer strain is a viable, already-validated alternative to the single-host *phaCAB* integration strategy pursued here. The two approaches carry different trade-offs: co-culture avoids the codon-compatibility and expression-burden constraints identified in the present computational design (Section 3.3 and Section 3.8), but introduces its own challenge of maintaining a stable population ratio between degrader and producer strains over extended cultivation, whereas the single-host strategy designed here, if experimentally validated, would avoid population-balance instability at the cost of the heterologous-expression burden documented throughout this study.

### 3.11. Circular Bioeconomy Implications of the Proposed CBP Platform

The successful computational design of *phaCAB* expression constructs in both *T. reesei* and *C. thermocellum* extends beyond cloning feasibility and provides insight into the potential development of integrated lignocellulosic biorefineries. HHs represent abundant agricultural waste that is frequently underutilised despite its high carbohydrate content. The proposed constructs establish a theoretical route through which cellulolytic microorganisms could convert lignocellulose-derived sugars into PHB, thereby transforming a low-value waste stream into a biodegradable polymer. Such an approach aligns with circular bioeconomy principles by promoting resource efficiency, reducing agricultural waste accumulation and supporting replacement of petroleum-derived plastics with renewable biomaterials.

## 4. Conclusions

This study developed an *in silico* framework for designing candidate PHB biosynthetic constructs for the cellulolytic hosts *T. reesei* and *C. thermocellum* within a broader hemp-hurd valorisation pathway. The *phaC*, *phaA* and *phaB* coding sequences were assessed individually for sequence integrity, predicted physicochemical characteristics and host-specific expression compatibility. Computational assembly, sequence alignment and simulated restriction analysis supported the internal consistency of the proposed constructs.

These findings demonstrate construct-level design feasibility only. They do not establish transformation, transcription, enzyme activity, PHB accumulation or direct conversion of HH biomass. Host-specific challenges include regulatory-sequence compatibility, codon usage, protein stability, acetyl-CoA and NADPH availability, metabolic burden, construct stability and competition between native metabolism and PHB biosynthesis. Experimental validation through host transformation, gene and protein expression analysis, quantitative PHB measurement, construct-stability testing and fermentation using hemp hurd-derived substrates is therefore required.

The principal contribution of this work is a comparative computational framework linking previous HH characterisation, pretreatment and hydrolysis studies with candidate PHB pathway designs for two mechanistically distinct cellulolytic hosts. The framework provides a defined starting point for the future development of consolidated bioprocessing systems for lignocellulosic biopolymer production.

## Figures and Tables

**Table 1:** Comparison of the present study with representative previous work on PHB production, host engineering and lignocellulosic conversion.

Category	Feedstock	Host	Engineering Approach	Experimental Outcome	Difference from Present Study	Representative Reference
Recombinant PHB production	Defined substrates	Recombinant bacterial host	PHB genes expressed	PHB experimentally quantified	Demonstrates expression but not hemp hurd-linked CBP	[22]
Lignocellulosic hydrolysate study	Fibre-reject hydrolysate	Recombinant *E. coli*	Separate hydrolysis and fermentation	PHB titre and cellular content measured	Experimental, but not cellulolytic CBP	[20]
Host expression study	Glucose or model medium	*T. reesei*	Host-specific expression cassette	Heterologous expression measured	Supports fungal cassette selection but not PHB production	[18]
Genetic-tool study	Model medium	*C. thermocellum*	*C. thermocellum* electrotransformation/genetic manipulation (pIKM1 itself sourced from Addgene plasmid #51054)	Electrotransformation demonstrated	Supports host-transformation feasibility but does not establish pIKM1 architecture or PHB-pathway expression	[19]
Present study	Hemp hurd valorisation framework	*T. reesei* and *C. thermocellum*	Host-comparative *in silico* PHB construct design	No experimental expression or PHB production	Links prior hemp-hurd processing work with candidate CBP constructs	This study

**Table 2:** Bioinformatic tools, vectors and sequence resources used for computational construct development.

Tool or Database	Version	Purpose in the Study	Input	Output Generated
NCBI GenBank	Online	Retrieval of the three separately extracted *phaC1*, *phaA* and *phaB1* CDSs	*Cupriavidus necator* H16 genome assembly GCA_000009285.2; *phaC1* H16_A1437, *phaA* H16_A1438 and *phaB1* H16_A1439	Reference nucleotide and CDS sequences
SnapGene	v6.2	*In silico* assembly, restriction simulation, virtual gels, ORF verification	Individual CDS; pET-24a(+); pIKM1; cassette parts	Construct maps, digestion profiles, virtual gels
Clustal Omega	v1.2.4	Sequence alignment and ORF integrity	CDS and translated PhaC, PhaA, PhaB	Per-gene alignment files
ExPASy ProtParam	Online	Physicochemical characterisation	Translated PhaC, PhaA, PhaB	MW, pI, instability, aliphatic index, GRAVY, extinction coefficient, estimated half-life
Biopython	v1.86	FASTA parsing, codon counting and CAI calculation	Individual stop-excluded CDSs; ribosomal-protein reference sets for *T. reesei* and *C. thermocellum*; Biopython SharpEcoliIndex for *E. coli*	Gene- and host-specific CAI values and codon-adaptiveness tables
Custom Python calculation (analysis script codon_analysis.py)	Python 3.11	ENC, GC%, ΔGC, rare-codon frequency and cluster analysis	phaC, phaA, phaB1 CDS FASTA files; host reference sets	Numerical codon-compatibility metrics and codon coordinates
OSTIR	v1.1	Predicted translation-initiation rate for each designed RBS	5′ leader/RBS sequence, start-codon context, first 99 nt of each CDS, anti-Shine–Dalgarno sequence	Predicted initiation rates (arbitrary units) for RBS1-RBS3
pET-24a(+)	5291 bp	Intermediate assembly in *E. coli* BL21(DE3)	BamHI and SalI	Intermediate construct (8984 bp)
pIKM1	6198 bp (Addgene depositor sequence)	Shuttle backbone for *C. thermocellum* construct	KflI and XbaI	pCtPHB1 construct: 6198 bp source vector; 6026 bp retained backbone after KflI/XbaI excision (10,169 bp final construct)
*tef1* promoter, *cbh1* terminator	—	Fungal expression regulatory parts	Cassette design	Three *T. reesei* expression cassettes
*groEL* promoter	—	Thermophile expression promoter	Cassette design	*C. thermocellum* expression cassette
*gpdA–hph–trpC*	~2.1 kb	Fungal selection (hygromycin B)	*T. reesei* construct	Selection cassette
Restriction enzymes	BamHI, SalI, XbaI, KflI	Directional assembly	Primer-incorporated sites	Compatible overhangs

**Table 3:** Detailed ExPASy ProtParam physicochemical characterisation of PhaA.

Parameter	Value
Protein name	PhaA (acetyl-CoA acetyltransferase)
Functional role	Condensation of two acetyl-CoA molecules to acetoacetyl-CoA
Amino-acid length	393 aa
Molecular weight	40.87 kDa
Theoretical isoelectric point (pI)	5.85
Total negatively charged residues (Asp + Glu)	44
Total positively charged residues (Arg + Lys)	36
Extinction coefficient (280 nm)	38,500 M^−1^·cm^−1^
Estimated half-life (*E. coli*, in vivo)	>10 h
Instability index	28.9 (Stable)
Aliphatic index	92.4
Grand average of hydropathicity (GRAVY)	−0.12

**Table 4:** Detailed ExPASy ProtParam physicochemical characterisation of PhaB.

Parameter	Value
Protein name	PhaB (acetoacetyl-CoA reductase)
Functional role	NADPH-dependent reduction of acetoacetyl-CoA
Amino-acid length	246 aa
Molecular weight	26.45 kDa
Theoretical isoelectric point (pI)	6.32
Total negatively charged residues (Asp + Glu)	28
Total positively charged residues (Arg + Lys)	27
Extinction coefficient (280 nm)	29,100 M^−1^·cm^−1^
Estimated half-life (*E. coli*, in vivo)	>10 h
Instability index	31.7 (Stable)
Aliphatic index	84.6
Grand average of hydropathicity (GRAVY)	−0.05

**Table 5:** Detailed ExPASy ProtParam physicochemical characterisation of PhaC.

Parameter	Value
Protein name	PhaC (polyhydroxybutyrate synthase)
Functional role	PHB chain elongation and polymerisation
Amino-acid length	589 aa
Molecular weight	63.98 kDa
Theoretical isoelectric point (pI)	6.61
Total negatively charged residues (Asp + Glu)	59
Total positively charged residues (Arg + Lys)	56
Extinction coefficient (280 nm)	68,200 M^−1^·cm^−1^
Estimated half-life (*E. coli*, in vivo)	>10 h
Instability index	35.4 (Stable)
Aliphatic index	88.1
Grand average of hydropathicity (GRAVY)	−0.09

**Table 6:** Gene-specific codon-usage and sequence-composition compatibility of the native *Cupriavidus necator* H16 *phaC*, *phaA* and *phaB* coding sequences with *E. coli* BL21 (DE3), *Clostridium thermocellum* DSM 1313 and *Trichoderma reesei* RUT C-30.

Gene	Host	CAI	ENC	Gene GC%	Host GC%	ΔGC	Rare Codons, n (%)	Rare-Codon Clusters
*phaC*	*E. coli* BL21(DE3)	0.7666	31.17	66.84	50.83	+16.00	0 (0.00%)	0
*phaA*	*E. coli* BL21(DE3)	0.7695	27.76	68.19	50.83	+17.36	0 (0.00%)	0
*phaB*	*E. coli* BL21(DE3)	0.7544	29.28	63.16	50.83	+12.33	0 (0.00%)	0
*phaC*	*C. thermocellum* DSM 1313	0.4832	31.17	66.84	39.15	+27.69	31 (5.26%)	2: codons 100–108 and 141–145
*phaA*	*C. thermocellum* DSM 1313	0.4912	27.76	68.19	39.15	+29.04	12 (3.05%)	0
*phaB*	*C. thermocellum* DSM 1313	0.4898	29.28	63.16	39.15	+24.01	10 (4.07%)	1: codons 93–102
*phaC*	*T. reesei* RUT C-30	0.6582	31.17	66.84	53.34	+13.50	4 (0.68%)	0
*phaA*	*T. reesei* RUT C-30	0.7356	27.76	68.19	53.34	+14.85	2 (0.51%)	0
*phaB*	*T. reesei* RUT C-30	0.7111	29.28	63.16	53.34	+9.82	0 (0.00%)	0

**Table 7:** Exact positions of host-defined rare-codon clusters identified in the native *C. necator* H16 *phaC* and *phaB* coding sequences relative to *C. thermocellum* DSM 1313 codon usage. Each reported range is the span from the first to the last rare codon within the qualifying 10-codon window (defined in Section 2.5) and may therefore be shorter than 10 codons; it is not the window itself.

Host	Gene	Cluster	Rare-Codon Span (First-to-Last, Within Qualifying Window)	Nucleotide Range	Rare-Codon Positions
*C. thermocellum* DSM 1313	*phaC*	1	100–108	298–324	100, 101, 108
*C. thermocellum* DSM 1313	*phaC*	2	141–145	421–435	141, 143, 145
*C. thermocellum* DSM 1313	*phaB*	1	93–102	277–306	93, 98, 102

**Table 8:** (a) Architecture of the host-specific *T. reesei* expression construct (TrePHB3 integration construct). Each biosynthetic gene forms an independent *tef1*-driven cassette. (b) Integration system for TrePHB3 integration construct. The homology arms are not part of the repeating tef1-Kozak-CDS-cbh1 cassette module shown in Table 8, but are physically part of the assembled construct and are therefore included in the 10,343 bp total.

(**a**)					
**Cassette**	**Promoter**	**5′ Kozak Context**	**Coding Sequence**	**Terminator**	**Size (bp)**
Cassette 1	*tef1*	CACC-ATG	*phaC* (1770 bp)	*cbh1*	~2620
Cassette 2	*tef1*	CACC-ATG	*phaA* (1182 bp)	*cbh1*	~2032
Cassette 3	*tef1*	CACC-ATG	*phaB* (741 bp)	*cbh1*	~1591
Selection	*gpdA*	—	*hph* (hygromycin B)	*trpC*	~2100
(**b**)					
**Component**	**Additional Component/Design Feature**				**Size (bp)**
Integration (NL1)	Candidate NL1 5′ and 3′ homology arms targeting the specified candidate integration region, designed to preserve the native *cbh1* locus				~2000

**Table 9:** Ribosome-binding site design for the *C. thermocellum* construct (pCtPHB1). Predicted translation-initiation rates were calculated with OSTIR using a host-specific anti-Shine–Dalgarno sequence; values are in arbitrary units on the RBS Calculator scale and are relative rather than absolute.

Ribosome-Binding Site (Gene)	Shine–Dalgarno (5′-3′)	Spacer (nt)	Predicted Initiation Rate (au)	Relative Strength	Role in Pathway Balancing	Unwanted Site
RBS1 (*phaC*)	AGAGGT	5	~3300	Moderate	Polymerises 3HB-CoA while limiting synthase burden	None
RBS2 (*phaA*)	AAGGAG	9	~10,200	Intermediate	Supplies acetoacetyl-CoA without excessive precursor drain	None
RBS3 (*phaB*)	AAGGAGG	7	~40,700	Strongest	Consumes acetoacetyl-CoA via NADPH-dependent reduction; is intended to reduce precursor build-up	None

**Table 10:** Proposed experimental validation roadmap for the candidate constructs. FTIR is included as a supporting identification method, not the sole quantitative PHB method.

Validation Stage	Method	Evidence Required
Construct assembly	Restriction analysis and whole-plasmid sequencing	Correct sequence, orientation and junctions
Host transformation	Fungal or anaerobic-bacterial transformation	Selectable or antibiotic-resistant transformants
Integration or retention	Junction PCR, plasmid rescue, qPCR or ddPCR	Correct integration or retained plasmid and copy number
Transcript analysis	RT-qPCR for all three genes	Transcription relative to housekeeping genes
Protein expression	Western blot, targeted proteomics or enzyme assays	*PhaC*, *PhaA* and *PhaB* detected
Enzyme function	Individual or pathway-level activity assays	Functional acetyl-CoA-to-PHB pathway
PHB screening	Nile red or BODIPY staining	Intracellular granules
PHB confirmation	GC-FID or GC-MS after methanolysis, supported by FTIR	Chemical confirmation and content
Process performance	Sugar consumption, titre, yield and productivity	Quantitative PHB production
Construct stability	Serial passage with and without selection	Stable genotype and phenotype
Hemp hurd validation	Hydrolysate followed by pretreated solids	Substrate utilisation and PHB production
CBP demonstration	Cellulose conversion and PHB formation in one system	Actual consolidated bioprocessing

**Figure A2:**
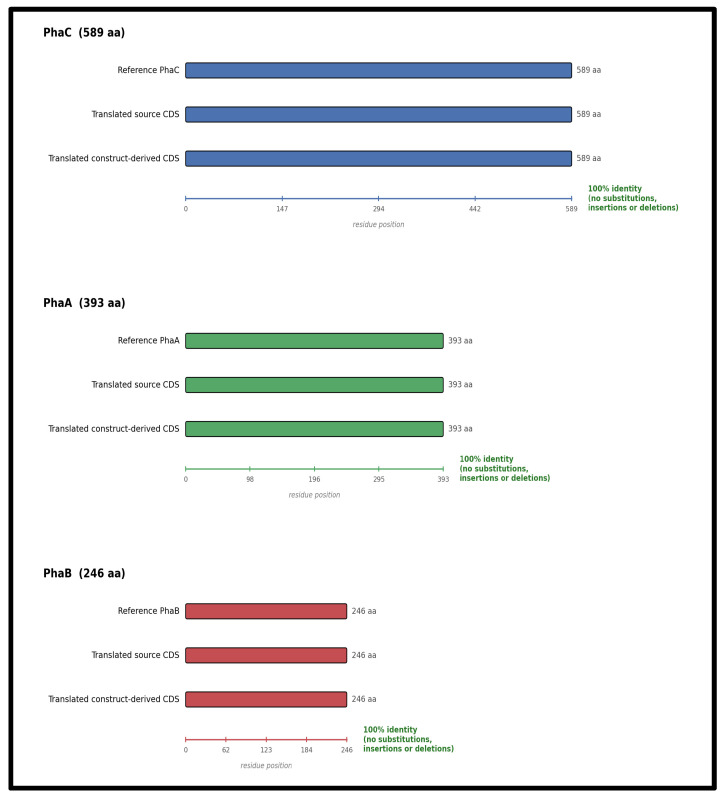
Construct-stage protein-level identity summary for phaC, phaA and phaB (translated source CDS vs. translated construct-derived CDS, amino-acid units), shown separately for each gene for reference purposes only. The three genes were analysed and handled as separate open reading frames throughout this study; this figure does not represent a fused PhaCAB protein.

## Data Availability

The sequence data used in this study are publicly available from the National Centre for Biotechnology Information (NCBI) under accession numbers GCA_000009285.2 (*Cupriavidus necator* H16), GCF_000184925.1 (*Clostridium thermocellum* DSM 1313), GCA_000513815.1 (*Trichoderma reesei* RUT C-30) and GCF_000009565.1 (*Escherichia coli* BL21 (DE3)). Additional computational analysis files (FASTA sequences, primer sequences, regulatory-part sequences, complete construct sequences in GenBank/SnapGene format, per-gene alignments, CAI/ENC/GC/rare-codon output, OSTIR input/output and exact digest-fragment tables) are available. Deposition in a persistent public repository is planned as a separate future action. The original contributions presented in this study are included in the article. Further inquiries can be directed to the corresponding author.

## Abbreviations

The following abbreviations are used in this manuscript:

aa	Amino acids
BL21(DE3)	*Escherichia coli BL21(DE3)*, used here as the intermediate assembly host
BODIPY	Boron-dipyrromethene fluorescent dye
bp	Base pairs
CAI	Codon adaptation index
CBM	Carbohydrate-binding module (CBM3 denotes family 3)
CBP	Consolidated bioprocessing
*C. necator*	*Cupriavidus necator*
*C. thermocellum*	*Clostridium thermocellum*
CDS	Coding sequence
ddPCR	Droplet digital polymerase chain reaction
DNA	Deoxyribonucleic acid
*E. coli*	*Escherichia coli*
ENC	Effective number of codons
FASTA	Text-based nucleotide or protein sequence format
FTIR	Fourier-transform infrared spectroscopy
GC	Guanine-cytosine content
GC-FID	Gas chromatography with flame-ionisation detection
GC-MS	Gas chromatography–mass spectrometry
GRAVY	Grand average of hydropathicity
HH	Hemp hurd
*hph*	Hygromycin B phosphotransferase gene/selectable marker
kb	Kilobases
kDa	Kilodaltons
LB	Lignocellulosic biomass
mRNA	Messenger RNA
MW	Molecular weight
NADH	Reduced nicotinamide adenine dinucleotide
NADPH	Reduced nicotinamide adenine dinucleotide phosphate
NCBI	National Center for Biotechnology Information
nt	Nucleotides
ORF	Open reading frame
PCR	Polymerase chain reaction
PHA	Polyhydroxyalkanoate
PHB	Polyhydroxybutyrate
pI	Isoelectric point
*phaCAB*	PHB biosynthetic gene cluster comprising phaC, phaA and phaB
PhaA	Acetyl-CoA acetyltransferase
PhaB	Acetoacetyl-CoA reductase
PhaC	Polyhydroxybutyrate synthase
qPCR	Quantitative polymerase chain reaction
RBS	Ribosome-binding site
RNA	Ribonucleic acid
RT-qPCR	Reverse-transcription quantitative polymerase chain reaction
SHF	Separate hydrolysis and fermentation
SSF	Simultaneous saccharification and fermentation
*T. reesei*	*Trichoderma reesei*
TCA	Tricarboxylic acid cycle
TIA	Technology Innovation Agency

## Appendix A

Appendix A presents schematic summaries of the separate protein-level identity checks for PhaC, PhaA and PhaB. Figure A1 shows the retrieval-stage identity summaries (reference versus translated sequence), referenced in Section 3.1 of the main text, and Figure A2 shows the construct-stage identity summaries for the same three genes.
